# Forensic application of CT-based frontal sinus and mandibular ramus measurements for sex and age estimation in a Turkish population: A retrospective analytical cross-sectional study

**DOI:** 10.1097/MD.0000000000047496

**Published:** 2026-01-30

**Authors:** Halil Kalkan, Emine Dursun Kalkan

**Affiliations:** a Department of Forensic Medicine, Gaziantep City Hospital, Gaziantep, Turkey; b Department of Forensic Medicine, Ağri Education and Research Hospital, Ağri, Turkey.

**Keywords:** age estimation, computed tomography, forensic anthropology, frontal sinus, mandibular ramus, ROC analysis, sex determination

## Abstract

Frontal sinus (FS) and mandibular ramus morphology are valuable components of forensic identification because these structures are resilient to trauma, fire, decomposition, and fragmentation, and they exhibit high individual variability. This study aimed to evaluate the diagnostic performance of computed tomography (CT)-based FS morphology and mandibular ramus length (MRL) for sex estimation and age-group classification. CT scans of 150 adults (75 males and 75 females) aged 20 to 72 years were retrospectively analyzed. Measurements included frontal sinus height, width, depth, volume, and MRL. Participants were stratified into 5 age groups. The individual and combined discriminative power of these parameters was assessed using multivariate statistical analysis, including discriminant analysis and receiver operating characteristic curve evaluation. All FS and mandibular ramus parameters were significantly higher in males than females (*P* < .001). Frontal sinus volume and MRL were the strongest sex predictors. The combined model demonstrated superior diagnostic performance (area under the curve = 0.91) compared with single-parameter analyses (area under the curve = 0.84–0.85), achieving 88% sensitivity, 85% specificity, and an overall accuracy of 86.5%. Frontal sinus depth increased with age (*P* = .031), whereas MRL decreased in older individuals (*P* = .012). Age classification accuracy was highest in the 20 to 29 (76.7%) and ≥60 (83.3%) age groups, with an overall accuracy of 72.0%. CT-based FS and mandibular ramus measurements provide reliable indicators for sex determination and offer moderate discriminative potential for age-group estimation. The integrated multivariate approach enhances classification accuracy and may be particularly useful in forensic scenarios where conventional skeletal markers are unavailable.

## 1. Introduction

In forensic sciences, identification not only aims to reveal the identity of an individual but also to determine key biological attributes such as sex, age, and, in some cases, ancestry. This process becomes particularly challenging in situations involving trauma, fire, decomposition, or dismemberment. Under such circumstances, the most robust and distinctive skeletal regions are preferred to construct a biological profile. The craniofacial region is frequently utilized in these analyses due to its protected anatomical structure and high degree of interindividual variability.^[1–3]^

Within this context, structures such as the frontal sinus (FS) and the mandibular ramus stand out as craniofacial elements with significant potential for both sex and age estimation.^[4]^ The FS begins to develop around the ages of 5 to 6 and typically reaches its final form by approximately 20 years of age.^[5,6]^ Due to the considerable variation in its size, shape, asymmetry, and degree of lobulation, the FS exhibits highly individual-specific morphology.^[7–9]^ Indeed, some researchers have referred to the FS as the “craniofacial fingerprint.”^[10–12]^ Studies using computed tomography (CT) have shown that this structure can be highly useful in sex differentiation.^[13–15]^

The mandible, the largest and strongest bone of the facial skeleton, is particularly notable for its ramus region, where sexual dimorphism is markedly evident. Parameters such as ramus height and length have consistently been reported to be greater in males than in females, and these differences are primarily attributed to hormonal influences and skeletal growth patterns.^[16–18]^ Additionally, the mandible undergoes age-related changes, including alveolar bone resorption, thinning at muscle attachment sites, and remodeling of the ramus.^[19]^

Today, CT is one of the most accurate imaging modalities used for morphological and metric evaluation of these structures.^[20]^ Techniques such as multiplanar reconstruction and 3D measurement allow for noninvasive, reproducible, and detailed analyses.^[21,22]^ These capabilities not only support the identification process in forensic cases but also facilitate the generation of data that can be scientifically analyzed. In addition to their forensic relevance, craniofacial morphometric markers derived from CT imaging also carry clinical and medico-legal importance. In trauma cases, particularly when conventional skeletal markers are destroyed or inaccessible, measurements of the FS and mandibular ramus can provide critical information for rapid identification and biological profiling. Such approaches are valuable for age estimation in medico-legal reporting, identification of dismembered or severely burned remains, and mass disaster victim identification. The reproducibility and noninvasive nature of CT techniques further highlight their potential for integration into routine forensic practice and clinical investigations requiring biological profiling.

Despite extensive evidence supporting the independent use of the FS and mandible, studies integrating both structures into a single analytical framework remain scarce. Most previous work has evaluated these parameters separately, leaving a gap in understanding their combined discriminative potential. Furthermore, few studies have provided comprehensive diagnostic performance metrics, such as receiver operating characteristic (ROC) analysis, sensitivity, specificity, and predictive values (measures that are increasingly emphasized in both forensic and clinical research).^[23–25]^

The present study addresses this gap by introducing one of the first integrated CT-based models that simultaneously evaluates FS and mandibular ramus morphometry for sex and age estimation. By applying multivariate discriminant analysis (MDA) and validating classification accuracy through ROC curves and confusion matrices, this study not only reinforces the discriminative power of these structures but also provides practical diagnostic insights. The findings may support forensic identification in challenging contexts and inform clinical practice in trauma and medico-legal investigations.

## 2. Methods

### 2.1. Study design and population

This study was designed as a retrospective and analytical cross-sectional investigation, allowing the assessment of significant differences and discriminative capacity across sex and age groups using inferential statistical methods. Ethical approval was obtained from the Clinical Research Ethics Committee of Gaziantep City Hospital (Approval No: 2025/246, Date: June 18, 2025). Since all data were retrospectively collected from the hospital’s digital archive system and contained no identifiable patient information, the study was conducted without the need for individual informed consent).

The final sample consisted of 150 eligible adults. This number reflects all CT scans that met the inclusion criteria during the study period (2023–2025). Because this was a retrospective forensic dataset, a predefined formula-based sample size calculation was not applicable. However, a post hoc power assessment demonstrated large effect sizes for key variables (Cohen *d* > 0.80), corresponding to a statistical power exceeding 0.90, indicating that the sample size was adequate for the analyses performed.

### 2.2. Sample selection and inclusion criteria

The study included adult individuals whose high-resolution brain and facial CT scans were archived between 2023 and 2025 at the Radiology Department of Gaziantep City Hospital. The inclusion criteria were as follows: individuals aged 20 years and above (to ensure complete FS development), verifiable sex information from medical records, and high-quality CT images with a slice thickness of 0.5 to 0.625 mm. Exclusion criteria included:

➢ A history of trauma, surgery, deformity, or tumor involving the FS or mandibular region.
➢ Hypoplasia or aplasia of the FS.
➢ CT images with significant artifacts that hindered reliable measurement.

During the initial screening, 18 CT scans were excluded due to motion artifacts (n = 7), FS aplasia or severe hypoplasia (n = 6), prior surgery or trauma affecting the craniofacial region (n = 3), or incomplete imaging fields (n = 2).

### 2.3. Imaging specifications

The CT images used in this study were obtained using multidetector computed tomography scanners. Specifically, the images were acquired using 64-slice GE VCT LightSpeed and 80-slice Canon Aquilion Prime SP devices. Imaging parameters were set as follows: tube voltage of 120 kV, tube current ranging from 45 to 175 mAs, slice thickness of 0.5 to 0.625 mm, field of view of 210 to 270 mm, and a matrix size of 512 × 512 pixels. All CT data were retrieved from the hospital’s Picture Archiving and Communication System. Subsequently, multiplanar reconstruction was performed to generate axial, coronal, and sagittal planes, and all measurements were conducted on these reconstructed images.

### 2.4. Measurement parameters and technique

All measurements were independently performed by a radiology-trained researcher and an experienced dentist. Each measurement was repeated twice, and intra-observer reliability was assessed using the intraclass correlation coefficient (ICC). An ICC value >0.90 was considered indicative of excellent agreement. To minimize measurement bias, both observers (a radiology-trained researcher and an experienced dentist) were blinded to the age and sex of the individuals during measurement extraction. In instances where anonymous picture archiving and communication system export did not fully mask demographic metadata, potential bias was mitigated by using predefined measurement protocols, independent duplicate measurements, and ICC validation to ensure objectivity.

**Figure 1.**
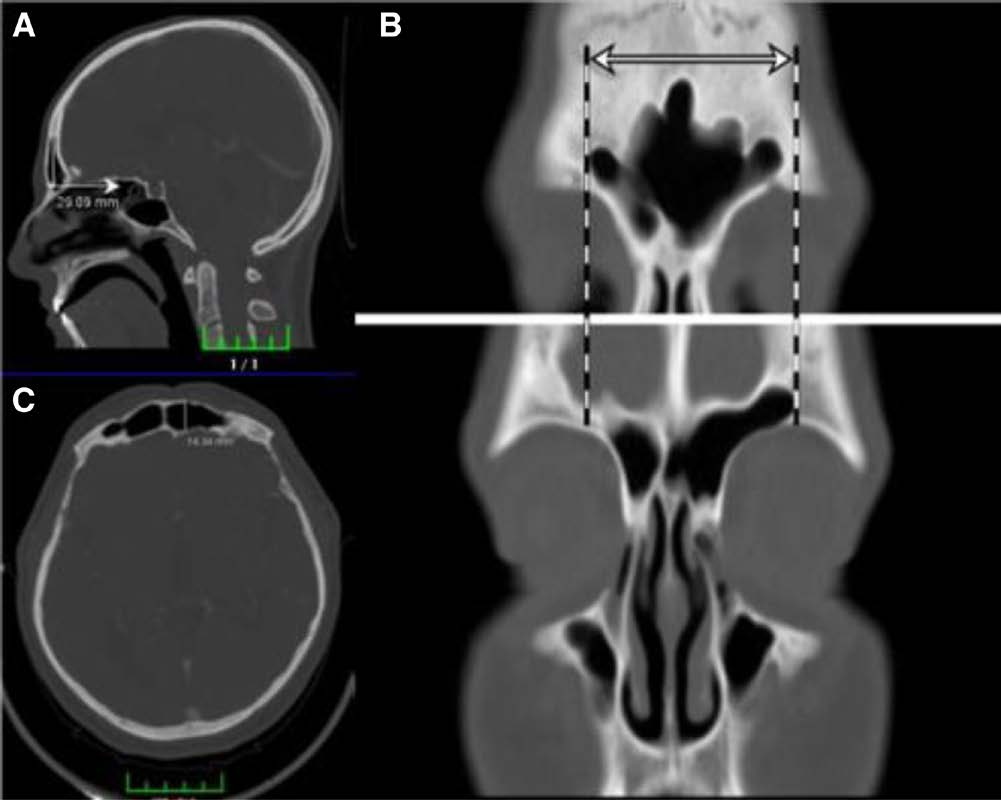
Demonstration of frontal sinus measurements on CT images. (A) Frontal sinus height (FSH), (B) frontal sinus width (FSW), (C) frontal sinus depth (FSD). CT = computed tomography.

FS measurements were conducted using CT slices obtained in the coronal and sagittal planes. In the coronal plane, frontal sinus height (FSH) was defined as the vertical distance from the highest to the lowest point of the sinus. In the same plane, frontal sinus width (FSW) was measured as the horizontal distance between the most lateral boundaries on the right and left sides. Frontal sinus depth (FSD) was assessed in the sagittal plane and defined as the maximum anteroposterior distance between the anterior and posterior walls of the sinus. All measurements were performed digitally on the CT workstation and recorded in millimeters. The frontal sinus volume (FSV) was estimated using a modified ellipsoid volume formula based on these 3 dimensions: volume ≈ (height × width × depth) × 0.523 (Fig. 1).

**Figure 2.**
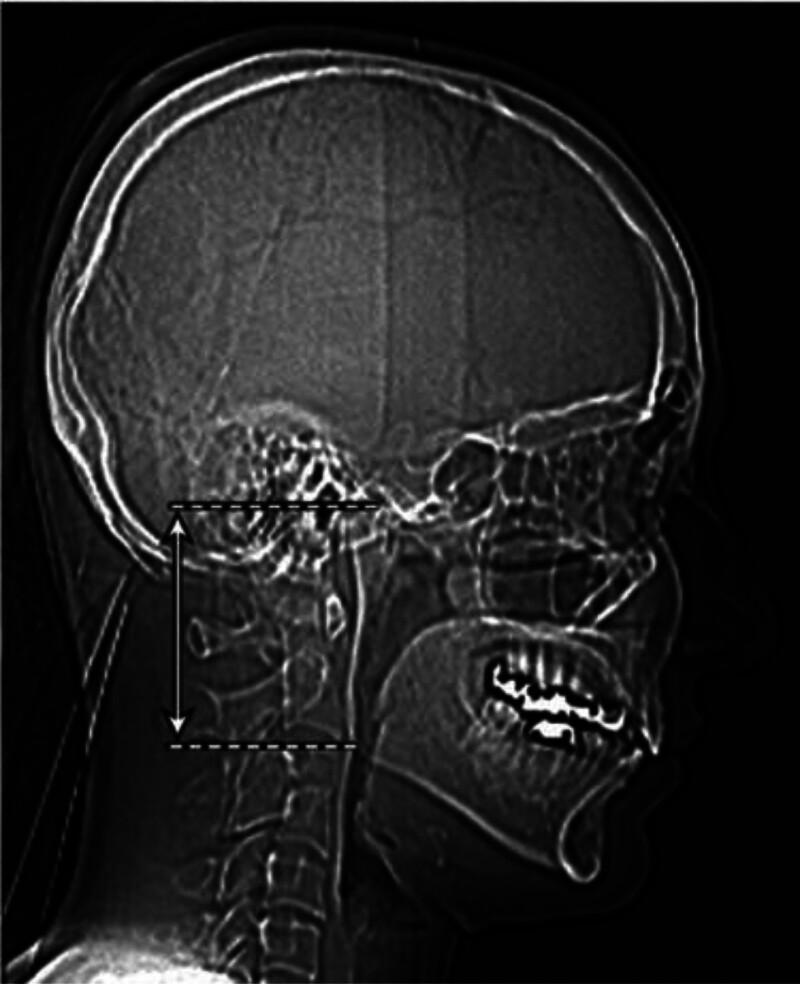
Demonstration of mandibular ramus length (MRL) measurement on a sagittal CT section. CT = computed tomography.

Mandibular ramus length (MRL) was measured on sagittal CT sections. The distance was defined as the straight line between the gonion point (representing the mandibular angle) and the condylion, the most superior point of the mandibular condyle (Fig. 2). All measurements were recorded digitally in millimeters and used in subsequent statistical analyses.

### 2.5. Age grouping

To evaluate morphological changes associated with age, the individuals included in the study were categorized into 5 age groups: 20 to 29, 30 to 39, 40 to 49, 50 to 59, and 60 years and older. This age stratification was designed to allow for a comparative analysis of age-related variability in both FS morphology and MRL.

### 2.6. Statistical analysis

Statistical analyses were performed using SPSS version 27.0 (IBM Corp., Armonk). Continuous variables were expressed as mean ± standard deviation, median, minimum, and maximum values. The Shapiro–Wilk test was used to evaluate normality of distribution. Differences between sexes were analyzed using the Student *t* test for normally distributed variables or the Mann–Whitney *U* test for non-normally distributed variables. Comparisons across age groups were conducted using one-way analysis of variance for normally distributed data and the Kruskal–Wallis test otherwise. To evaluate classification performance for sex and age estimation, MDA was applied. Model performance was assessed using Wilks’ Lambda, eigenvalue, canonical correlation coefficient, and the Chi-square test. In addition, diagnostic accuracy was further examined using ROC curve analysis, with calculation of the area under the curve (AUC), sensitivity, specificity, positive predictive value, and negative predictive value. A confusion matrix was generated to assess classification performance by sex. Statistical significance was defined as a *P*-value <.05.

## 3. Results

Descriptive statistics for FS and mandibular ramus measurements of the 150 individuals included in the study revealed a wide range of variation across all parameters. The mean FSH was 31.2 ± 4.8 mm, with a minimum of 22.0 mm, a maximum of 41.5 mm, and a median of 31.0 mm. The mean FSW was 39.2 ± 6.1 mm, while the FSD measured 16.2 ± 2.9 mm on average. Based on these three-dimensional values, the FSV was calculated to have a mean of 10.9 ± 3.4 cm^3^, ranging from 4.2 cm^3^ to 18.9 cm^3^. The MRL was found to have a mean value of 57.5 ± 6.2 mm, with minimum and maximum values of 42.0 mm and 71.3 mm, respectively (Table 1).

**Table 1:** Descriptive statistics of frontal sinus and mandibular ramus measurements (N = 150).

Parameter	Mean ± SD	Median (min–max)
Frontal sinus height (FSH) (mm)	31.2 ± 4.8	31.0 (22.0–41.5)
Frontal sinus width (FSW) (mm)	39.2 ± 6.1	39.0 (25.0–53.0)
Frontal sinus depth (FSD) (mm)	16.2 ± 2.9	16.0 (10.5–22.8)
Frontal sinus volume (FSV) (cm^3^)	10.9 ± 3.4	10.7 (4.2–18.9)
Mandibular ramus length (MRL) (mm)	57.5 ± 6.2	57.0 (42.0–71.3)

SD = standard deviation.

Sex-based comparisons of FS and mandibular ramus measurements are presented in Table 2. In all measured parameters, male individuals exhibited significantly higher mean values compared to females (*P* < .001). The mean FSH was 33.8 ± 4.2 mm in males and 28.6 ± 3.7 mm in females. Similarly, FSW was measured as 42.1 ± 5.6 mm in males and 36.2 ± 4.9 mm in females; FSD was 17.4 ± 2.8 mm in males versus 14.9 ± 2.3 mm in females. Based on these 3 dimensions, the calculated FSV was significantly greater in males (12.8 ± 3.1 cm^3^) compared to females (9.1 ± 2.7 cm^3^). Additionally, MRL was also significantly longer in males, with a mean of 61.3 ± 5.2 mm, compared to 53.7 ± 4.8 mm in females (Table 2).

**Table 2:** Sex-based comparison of frontal sinus and mandibular ramus measurements.

Parameter	Male (n = 75)	Female (n = 75)	*P*-value
Frontal sinus height (FSH) (mm)	33.8 ± 4.2	28.6 ± 3.7	<.001*
Frontal sinus width (FSW) (mm)	42.1 ± 5.6	36.2 ± 4.9	<.001*
Frontal sinus depth (FSD) (mm)	17.4 ± 2.8	14.9 ± 2.3	<.001*
Frontal sinus volume (FSV) (cm^3^)	12.8 ± 3.1	9.1 ± 2.7	<.001*
Mandibular ramus length (MRL) (mm)	61.3 ± 5.2	53.7 ± 4.8	<.001*

* Student *t* test.

Comparisons of FS and mandibular ramus measurements across age groups are presented in Table 3. No statistically significant differences were observed between age groups in FSH, width (FSW), or volume (FSV) parameters (*P* > .05). In contrast, FSD showed a statistically significant difference across age groups (*P* = .031). The lowest values were recorded in the 20 to 29 age group, while the highest values were observed in individuals aged 60 and above. A significant age-related difference was also found in MRL (*P* = .012). The highest mean MRL was measured in the 20 to 29 age group (59.1 ± 5.9 mm), with a decreasing trend observed in older age groups. In individuals aged 60 and above, the mean MRL was 56.0 ± 6.7 mm (Table 3).

**Table 3:** Comparisons of frontal sinus and mandibular ramus measurements across age groups.

Parameter	20–29 yr (n = 30)	30–39 yr (n = 30)	40–49 yr (n = 30)	50–59 yr (n = 30)	60+ yr (n = 30)	*P*-value
Frontal sinus height (FSH) (mm)	30.8 ± 4.5	31.4 ± 4.2	31.6 ± 5.0	31.0 ± 4.9	31.2 ± 5.1	.824*
Frontal sinus width (FSW) (mm)	39.1 ± 6.0	39.6 ± 6.2	39.3 ± 6.3	39.0 ± 6.5	39.0 ± 6.1	.962*
Frontal sinus depth (FSD) (mm)	15.3 ± 2.5	15.8 ± 2.7	16.3 ± 2.9	16.7 ± 3.0	17.0 ± 3.1	.031*
Frontal sinus volume (FSV) (cm^3^)	10.1 ± 3.0	10.6 ± 3.2	11.1 ± 3.3	11.4 ± 3.5	11.3 ± 3.6	.108*
Mandibular ramus length (MRL) (mm)	59.1 ± 5.9	58.4 ± 6.2	57.1 ± 6.1	56.2 ± 6.4	56.0 ± 6.7	.012†

ANOVA = analysis of variance.

* Kruskal–Wallis test.

† One-way ANOVA.

The results of the discriminant analysis based on FS and mandibular ramus parameters are presented in Table 4. A MDA using these measurements produced a classification model capable of accurately predicting sex. All parameters included in the model made statistically significant contributions to sex differentiation. According to the structure coefficients, the most discriminative variable was FSV (SC = 0.731), followed by MRL (SC = 0.685), FSW (SC = 0.591), and FSH (SC = 0.487). The lowest structure coefficient was observed for FSD (SC = 0.405). When evaluating the standardized discriminant function coefficients by sex, all variables had higher weights in the male group, suggesting stronger discriminative contributions in males. Notably, FSV (male: 2.834; female: 2.217) and MRL (male: 1.542; female: 1.173) were the most influential variables in distinguishing sex. The constant terms for the discriminant functions were calculated as -115.40 for males and -97.35 for females (Table 4).

**Table 4:** Discriminant analysis results based on frontal sinus and mandibular ramus parameters.

Parameter	Structure coefficient (SC)	Male function coefficient	Female function coefficient
Frontal sinus volume (FSV) (cm^3^)	0.731	2.834	2.217
Mandibular ramus length (MRL) (mm)	0.685	1.542	1.173
Frontal sinus width (FSW) (mm)	0.591	1.209	0.918
Frontal sinus height (FSH) (mm)	0.487	0.875	0.743
Frontal sinus depth (FSD) (mm)	0.405	0.663	0.538
Constant	–	-115.40	-97.35

Model performance indicators			
Indicator		Value	
Wilks’ Lambda		0.412	
Eigenvalue		1.608	
Canonical correlation		0.782	
Chi-square		189.32	
*P*-value		<.001	
Overall classification accuracy		86.5%	
Male classification accuracy		85.0%	
Female classification accuracy		88.0%	

The statistical validity of the model was found to be robust. The Wilks’ Lambda value was 0.412, the canonical correlation coefficient was 0.782, the eigenvalue was 1.608, and the Chi-square value was 189.32 (*P* < .001). The classification accuracy was calculated as 85.0% for males, 88.0% for females, and 86.5% overall (Table 4).

The classification accuracy according to age groups is presented in Table 5. Based on the results of the MDA for age estimation, the overall classification accuracy was determined to be 72.0%. The model’s ability to differentiate between age groups varied across the cohorts. The highest classification accuracy was observed in the 60+ age group, with 83.3% of individuals (n = 25) correctly classified. This was followed by the 20 to 29 age group with 76.7% accuracy (n = 23) and the 50 to 59 age group with 70.0% accuracy (n = 21). In contrast, the classification performance was comparatively lower in the middle age groups. The 30 to 39 age group had a correct classification rate of 66.7% (n = 20), while the 40 to 49 age group had the lowest at 63.3% (n = 19) (Table 5).

**Table 5:** Classification accuracy by age group (results of discriminant analysis, n = 150).

Actual age group	20–29	30–39	40–49	50–59	60+	Total (n)	Correct classification (n)	Accuracy (%)
20–29	23	4	2	1	0	30	23	76.7%
30–39	3	20	4	2	1	30	20	66.7%
40–49	2	3	19	4	2	30	19	63.3%
50–59	1	3	2	21	3	30	21	70.0%
60+	0	1	2	2	25	30	25	83.3%
Total	–	–	–	–	–	150	108	72.0%

**Figure 3.**
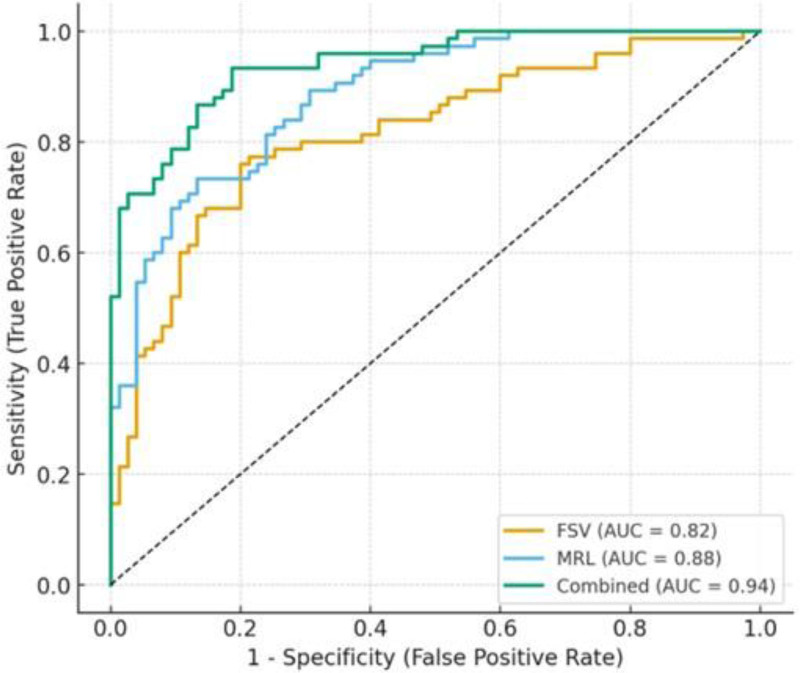
ROC curves for sex classification. ROC = receiver operating characteristic.

Performance metrics of discriminant analysis model for sex estimation was shown in Table 6. ROC analysis revealed that the FSV achieved an AUC of 0.85, while the MRL yielded an AUC of 0.84. The combined model (FSV + MRL + other parameters) demonstrated the highest discriminative power with an AUC of 0.91. For sex classification, the model achieved a sensitivity of 88%, specificity of 85%, and an overall accuracy of 86.5%. Positive predictive value and negative predictive value were 87% and 86%, respectively (Table 6 and Fig. 3).

**Table 6:** Performance metrics of discriminant analysis model for sex estimation.

Parameter	AUC (95% CI)	Sensitivity (%)	Specificity (%)	Accuracy (%)	PPV (%)	NPV (%)
Frontal sinus volume (FSV)	0.85 (0.78–0.90)	82.0	80.0	81.0	83.0	79.0
Mandibular ramus length (MRL)	0.84 (0.77–0.89)	80.0	81.0	80.5	82.5	78.5
Combined model (FSV + MRL + others)	0.91 (0.86–0.95)	88.0	85.0	86.5	87.0	86.0

AUC = area under the curve, MRL = mandibular ramus length, NPV = negative predictive value, PPV = positive predictive value.

**Figure 4.**
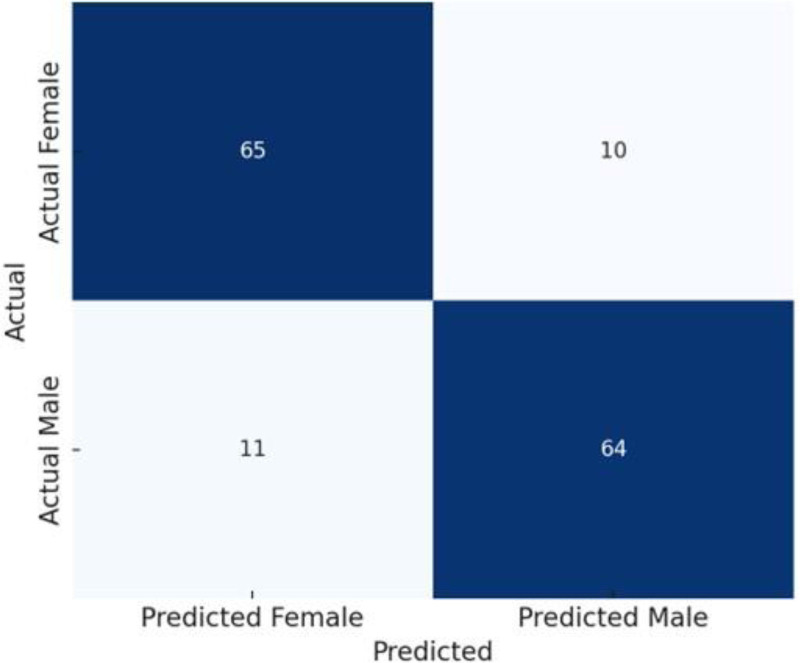
Confusion matrix (combined model).

Confusion matrix of discriminant model for sex classification was shown in Table 7. The model correctly classified 64 out of 75 males (85.3%) and 66 out of 75 females (88.0%). Overall, 130 out of 150 individuals were correctly classified, corresponding to an accuracy of 86.5% (Table 7 and Fig. 4).

**Table 7:** Confusion matrix of the discriminant model for sex classification (N = 150).

	Predicted male	Predicted female	Total
Actual male	64	11	75
Actual female	9	66	75
Total	73	77	150

**Figure 5.**
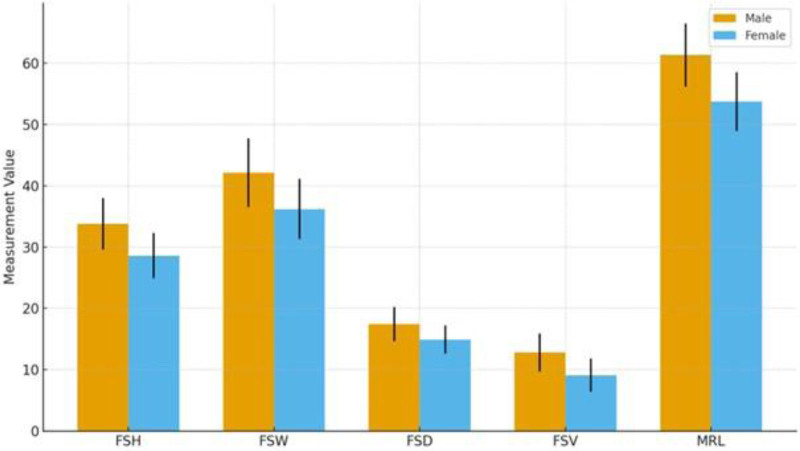
Sex-based comparison of frontal sinus and mandibular ramus measurements.

Figure 5 was demonstrated that males have consistently higher values for FSH, width (FSW), depth (FSD), volume (FSV), and MRL, with visible separation between groups and nonoverlapping confidence intervals, supporting statistically significant sex differences (all *P* < .001) (Fig. 5).

**Figure 6.**
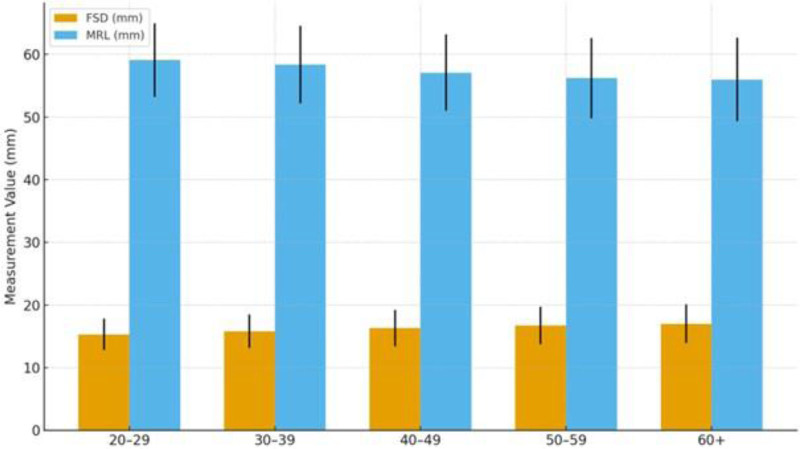
Age-group comparison of frontal sinus depth and mandibular ramus length.

Figure 6 was demonstrated that the progressive increase in FSD across age groups, with the highest values observed in individuals aged ≥60 years. Conversely, MRL shows a gradual decline from younger to older age categories. These visual trends align with the statistical findings indicating significant age-related morphological changes (FSD: *P* = .031; MRL: *P* = .012) (Fig. 6).

## 4. Discussion

Recent forensic research has emphasized the diagnostic utility of craniofacial structures, particularly the FS and mandible, due to their resistance to trauma, fire, and postmortem degradation.^[26–29]^ Previous studies have independently shown that FS morphology exhibits substantial interindividual variability and sexual dimorphism, with males typically demonstrating larger dimensions and greater sinus pneumatization.^[13–15]^ Investigations of the mandibular ramus have consistently documented significant sex differences in ramus height, gonial angle, and overall ramus dimensions.^[28,29]^ In line with these reports, the present study confirms that both anatomical regions remain strong contributors to sex estimation. However, our integrated approach extends the literature by combining sinus and ramus metrics within a single discriminant framework, enabling higher classification accuracy than studies examining each structure separately.

Anatomically, the FS is considered a highly individual-specific structure, making it a valuable feature in identification, especially in cases where the craniofacial region remains intact and unaffected by trauma or decomposition. Hekimoglu et al analyzed FS dimensions using CT images and reported that both volume and linear measurements were significantly higher in males, allowing for sex differentiation with an accuracy rate of 76%.^[28]^ Patel et al highlighted the uniqueness of FS morphology and noted that height and width measurements were particularly reflective of sex differences.^[29]^ Consistent with previous studies, our findings also showed that FS height, width, depth, and volume were all significantly higher in males than in females (*P* < .001).

The mandibular ramus region is highly responsive to both functional loading and hormonal influences, making it one of the most sexually dimorphic craniofacial structures.^[18]^ Franklin et al emphasized that mandibular morphology is a reliable indicator for sex estimation and that ramus height is significantly greater in males.^[27]^ Stipo et al demonstrated the utility of the mandible in sex determination and noted that changes in ramus morphology are influenced by growth, hormonal effects, and functional adaptation.^[26]^ These findings from the literature are consistent with our results, which revealed a significantly greater MRL in males compared to females.

In terms of age estimation, our study found that FSD increased gradually with age, while MRL decreased over time. In particular, individuals aged 60 and above exhibited the greatest FSD and the shortest MRL. These findings suggest that age-related changes in bone resorption and sinus pneumatization affect craniofacial morphology. Gawlikowska-Sroka et al reported notable bone resorption in the mandible with aging, along with a reduction in both ramus height and length.^[30]^ Additionally, Sidhu et al., in a study evaluating FS and mandibular morphometry together, stated that age exerts measurable effects on both structures and that their combined use can enhance age classification accuracy.^[31]^ Our findings support this integrative approach, with the most accurate age classification observed in the 20 to 29 and 60+ year groups, further confirming the diagnostic value of these anatomical features when assessed together.

Prior studies have used different methodological strategies to evaluate FS morphology. Several authors relied on linear measurements (height, width, and anteroposterior depth) derived from coronal and sagittal CT slices, which provide reliable and reproducible dimensions.^[13,27]^ Other studies applied volumetric assessment, using semiautomatic segmentation or ellipsoid volume approximations to quantify sinus capacity, demonstrating higher discriminative potential for sex estimation.^[26]^ Furthermore, cone-beam CT studies employed three-dimensional reconstruction and manual tracing of sinus boundaries to assess asymmetry, lobulation, and sinus contour complexity.^[14,15]^ These methodological differences are important for interpreting variability in classification performance across studies. In our study, the modified ellipsoid formula using 3 orthogonal dimensions ensured consistency, reproducibility, and comparability with both linear and volumetric approaches described in earlier work.

From a practical standpoint, the present findings offer clear implications for both forensic and clinical applications. In forensic contexts, the integrated CT-based model can assist in challenging identification scenarios, including fire-related fatalities, advanced decomposition, or fragmented remains, where conventional skeletal elements may be unavailable. Clinically, these morphometric markers may support trauma-related identification, contribute to medico-legal age estimation in official reporting, and provide objective parameters in mass disaster victim identification. Furthermore, the integration of FS and mandibular ramus measurements into AI-based automated classification systems represents a promising future direction, potentially enabling rapid, accurate, and reproducible sex and age estimation across diverse forensic and clinical settings.

Previous research has largely focused on either the FS or the mandible as independent predictors of biological identity. While these structures individually show discriminative potential, studies integrating both regions within a unified analytical framework remain scarce. Our study provides one of the first CT-based models that simultaneously evaluates FS morphometry and MRL for both sex and age estimation. By combining 2 craniofacial elements with distinct developmental and age-related characteristics, the present model achieved higher classification accuracy compared to prior single-structure approaches. This integrated methodology underscores the added value of multivariate analysis in forensic anthropology.

Hekimoğlu et al recently demonstrated the utility of paranasal sinus volumes in sex estimation, reporting accuracies around 76%, whereas Patel et al emphasized the discriminative role of sinus height and width.^[28,29]^ Franklin and colleagues highlighted mandibular dimensions as robust indicators of sexual dimorphism. However, these studies evaluated each structure in isolation.^[27]^ By contrast, our combined model increased sex classification accuracy to 86.5% and allowed moderate age differentiation, particularly at the youngest and oldest extremes. Although direct studies integrating multiple craniofacial structures for combined sex and age estimation are limited, related research provides supportive context: for instance, Sikaria et al highlighted the gonial angle as a key craniofacial landmark for both age and sex determination.^[32]^ Suzuki et al demonstrated significant correlations between Hounsfield unit values of the mandibular condyle and palate and age, and formulated a predictive age estimation model.^[33]^ These findings align with our integrated CT-based approach and underscore the potential advantages of multistructural analysis for forensic applications.

## 5. Limitations

This study has some limitations that should be acknowledged. First, the sample was derived from a single-center hospital population, which may restrict the generalizability of the results to other ethnic and geographic groups. Craniofacial morphology is known to exhibit population-specific variation, and future multicenter studies with diverse cohorts are warranted to validate our findings. Second, the retrospective cross-sectional design limited the ability to establish causal inferences regarding age-related changes. Third, although our integrated model achieved high accuracy for sex estimation, the classification accuracy for age was moderate in middle-aged groups, reflecting the gradual and less distinct morphological changes in this life stage. Fourth, the study did not perform external validation of the discriminant functions, which will be critical for ensuring reproducibility in independent datasets. Finally, while our approach focused on linear and volumetric CT measurements, advanced techniques such as three-dimensional reconstruction, geometric morphometrics, and machine learning algorithms could further enhance predictive performance. Despite these limitations, our study represents one of the first attempts to integrate FS and mandibular ramus parameters into a unified CT-based model, highlighting its novelty and potential as a reproducible tool for forensic and clinical applications.

## 6. Conclusions

In conclusion, the findings demonstrated that both anatomical structures exhibited high discriminative power for sex determination and were particularly effective in classifying individuals at the extremes of the age spectrum. FS and mandibular ramus measurements obtained via CT imaging provide reliable and reproducible indicators for sex estimation and have moderate potential for age classification. Our study provides one of the first integrated CT-based models that combines FS and mandibular ramus metrics for simultaneous sex and age estimation, highlighting its novelty compared to prior single-structure approaches. This integrated approach enhances classification performance and offers practical applicability in forensic identification, particularly in challenging scenarios where conventional skeletal elements are unavailable. Moreover, these findings may serve as a basis for the future development of AI-assisted automated classification systems, further strengthening their forensic and clinical utility.

## Author contributions

**Conceptualization:** Halil Kalkan.

**Data curation:** Halil Kalkan, Emine Dursun Kalkan.

**Formal analysis:** Halil Kalkan, Emine Dursun Kalkan.

**Funding acquisition:** Halil Kalkan, Emine Dursun Kalkan.

**Investigation:** Halil Kalkan, Emine Dursun Kalkan.

**Methodology:** Halil Kalkan, Emine Dursun Kalkan.

**Resources:** Halil Kalkan, Emine Dursun Kalkan.

**Software:** Halil Kalkan, Emine Dursun Kalkan.

**Validation:** Halil Kalkan, Emine Dursun Kalkan.

**Visualization:** Halil Kalkan.

**Writing – original draft:** Halil Kalkan.

**Writing – review & editing:** Emine Dursun Kalkan.
